# Resveratrol-Mediated Attenuation of *Staphylococcus aureus* Enterotoxin B-Induced Acute Liver Injury Is Associated With Regulation of microRNA and Induction of Myeloid-Derived Suppressor Cells

**DOI:** 10.3389/fmicb.2018.02910

**Published:** 2018-12-17

**Authors:** Sabah Kadhim, Narendra P. Singh, Elizabeth E. Zumbrun, Taixing Cui, Saurabh Chatterjee, Lorne Hofseth, Abduladheem Abood, Prakash Nagarkatti, Mitzi Nagarkatti

**Affiliations:** ^1^Department of Pathology, Microbiology and Immunology, University of South Carolina School of Medicine, Columbia, SC, United States; ^2^Department of Cell Biology and Anatomy, University of South Carolina School of Medicine, Columbia, SC, United States; ^3^Environmental Health and Disease Laboratory, Department of Environmental Health Sciences, Arnold School of Public Health, University of South Carolina, Columbia, SC, United States; ^4^Department of Drug Discovery and Biomedical Sciences, South Carolina College of Pharmacy, University of South Carolina, Columbia, SC, United States; ^5^College of Dental Medicine, Al-Mustansiriya University, Baghdad, Iraq

**Keywords:** resveratrol, *Staphylococcus aureus* enterotoxin B, acute liver injury, microRNA, myeloid derived suppressor cells

## Abstract

Resveratrol (RES) is a polyphenolic compound found abundantly in plant products including red grapes, peanuts, and mulberries. Because of potent anti-inflammatory properties of RES, we investigated whether RES can protect from Staphylococcal enterotoxin B (SEB)-induced acute liver injury in mice. SEB is a potent super antigen that induces robust inflammation and releases inflammatory cytokines that can be fatal. We observed that SEB caused acute liver injury in mice with increases in enzyme aspartate transaminase (AST) levels, and massive infiltration of immune cells into the liver. Treatment with RES (100 mg/kg body weight) attenuated SEB-induced acute liver injury, as indicated by decreased AST levels and cellular infiltration in the liver. Interestingly, RES treatment increased the number of myeloid derived suppressor cells (MDSCs) in the liver. RES treatment led to alterations in the microRNA (miR) profile in liver mononuclear cells (MNCs) of mice exposed to SEB, and pathway analysis indicated these miRs targeted many inflammatory pathways. Of these, we identified miR-185, which was down-regulated by RES, to specifically target Colony Stimulating Factor (CSF1) using transfection studies. Moreover, the levels of CSF1 were significantly increased in RES-treated SEB mice. Because CSF1 is critical in MDSC induction, our studies suggest that RES may induce MDSCs by down-regulating miR-185 leading to increase the expression of CSF1. The data presented demonstrate for the first time that RES can effectively attenuates SEB-induced acute liver injury and that this may result from its action on miRs and induction of MDSCs.

## Introduction

Resveratrol (RES: *trans*-3,5,4′-trihydroxystilbene) is a non-flavonoid polyphenolic compound found abundantly in a large number of plant products including red grapes, red wine, peanut, mulberries and the like (Jang et al., 1997; Jang and Surh, 2001). RES is produced as a part of a plant’s defense system against fungal infection and is a member of the class of plant antibiotic compounds (Soleas et al., 1997a,b). Most importantly, RES was an essential component of Ko-jo-kon, an oriental medicine, used to treat diseases of the blood vessels, heart (Soleas et al., 1997a,b), and liver (Soleas et al., 1997a,b). In recent years, RES has been the focus of many studies, including our own laboratory, for its pharmacological and beneficial properties on a wide range of diseases, including cardiovascular, autoimmune, neurological, and hepatic (Baur and Sinclair, 2006; Singh et al., 2007a, 2011). We have previously shown the beneficial effects of RES on autoimmune and inflammatory diseases (Singh et al., 2007a, 2010, 2011; Singh U.P. et al., 2012). In addition, RES also has antibacterial activity. Ma et al. (2018) recently described the potential antibacterial activity of RES against foodborne pathogens, its mechanisms of action, and its possible applications in food packing and processing. We have also demonstrated the protective effect of RES against Staphylococcal enterotoxin B-induced lung injury (Rieder et al., 2012; Alghetaa et al., 2018). In another study, Yang and Lim showed that RES ameliorates hepatic metaflammation, accompanied by alterations in NLRP3 inflammasome (Yang and Lim, 2014). Due to the wide-ranging beneficial effects of RES, it has recently been introduced as a nutritional supplement in the market (Aschemann-Witzel and Grunert, 2015) and presently, it is being used extensively, not only in United States but also worldwide.

Staphylococcal enterotoxin B (SEB) is a well-known super antigen that is highly toxic. SEB bypasses the normal antigen processing by antigen-presenting cells (APCs). It interacts outside of the peptide-binding groove of major histocompatibility complex class II (MHC II) molecule, with certain external Vβ domains located on T cell receptor (TCR) to activate such T cells and cause cytokine storm (Marrack and Kappler, 1990; Baker and Acharya, 2004). While normal antigens activate approximately 0.1% of host T cells, super antigens, on the other hand, can activate up to 30% of host T cells (Rieder et al., 2011). Such massive T cell activation leads to uncontrolled proinflammatory cytokine release, termed as a cytokine storm, including the release of tumor necrosis factor-alpha (TNF-α), interferon-gamma (IFN-γ), and interleukins IL-1, IL-2, IL-6, IL-8, and IL-12 (Miethke et al., 1992; Pfeffer et al., 1993; Krakauer et al., 2010). Despite improvements in healthcare, *Staphylococcus aureus* (*S*. *aureus*) exposure still results in 20–30% mortality in the developed world, partly because of the ability of this bacterium to acquire antibiotic resistance (Chambers and Deleo, 2009). *S*. *aureus* secretes major virulence factor that causes community acquired diseases and nosocomial infections (Dinges et al., 2000; Pinchuk et al., 2010). Also, SEB exposure in humans can cause severe food poisoning and sometimes, it can cause even fatal conditions including toxic shock syndrome (Henghold, 2004). SEB is an extremely stable compound in acidic environments (gastrointestinal tract) and is highly resistant to heat and proteolytic digestion (Ler et al., 2006). Because of such toxicity and ability to cause death, it has the potential to be used as a biological weapon, and to that end, Centers for Disease Control (CDC) has classified SEB as a category B priority agent (Madsen, 2001). Currently, there is no effective treatment to prevent SEB-mediated toxicity and thus, there is a dire need for a more effective treatment modality to control rapid T cell activation and cytokine storm induced by SEB.

Whether RES, which has potent anti-inflammatory properties, can effectively protect the liver from SEB-induced acute liver injury acute liver injury has not been previously studied. In this study, we demonstrate that RES protects mice from acute liver injury. Moreover, this protection was associated with altered expression of microRNA and induction of MDSCs. Specifically, we found that miR-130a and miR-185 directly target CSF1 [also known as macrophage colony stimulating factor (M-CSF)] gene in liver MNCs, which plays a critical role in the induction of MDSCs. The present study demonstrates that RES protects mice against SEB-induced acute liver injury possibly through regulation of microRNA to induce immunosuppressive MDSCs.

## Materials and Methods

### Animals

C57Bl/6 female mice were obtained from Jackson laboratory. The Institutional Animal Care and Use Committee (IACUC) of University of South Carolina approved the protocol and use of mice. The mice were housed in a pathogen-free AALAC approved animal facility at University of South Carolina School of Medicine.

### Chemicals and Reagents

The following chemicals were purchased and used: SEB (Toxin Technologies, Sarasota, FL, United States), RES and DMSO (Sigma-Aldrich, St. Louis, MO, United States), culture medium (RPMI 1640), Penicillin/Streptomycin, HEPES, L-glutamine, FBS, and PBS (Invitrogen Life Technologies, Carlsbad, CA, United States). Fluorophore-labeled anti-mouse CD3, CD4, CD8, CD44, NK1.1, CD11b, and Gr1 antibodies were purchased from eBioScience (Carlsbad, CA, United States). Bio-Plex kit for mouse cytokines was purchased from Bio-Rad (Bio-Rad, Hercules, CA, United States). Polymerase chain reaction (PCR) reagents, Epicentre’s PCR premix F and Platinum *Taq* Polymerase, were purchased form Invitrogen Life Technologies (Carlsbad, CA, United States). miRNeasy kit, miScript cDNA synthesis kit, miScript primer assays kit, miScript SYBR Green PCR kit, miR-185-5P mimic, and miR-185-5p inhibitor, SsoAdvanced SYBR green supermix from Bio-Rad (Hercules, CA, United States) were purchased from QIAGEN (Qiagen, Inc., Valencia, CA, United States).

### SEB-Induced Acute Liver Injury and RES Treatment

We tested the efficacy of RES in an *in vivo* mouse model of acute liver injury induced by SEB. To that end, SEB was injected intraperitonally (i.p.) into C57BL/6 mice at a dose of 40 μg in PBS, as described previously (Rieder et al., 2011; Rao et al., 2014). The mice were first sensitized by injecting (i.p.) D-galactosamine (Dgal; 20 mg) in PBS 30 min prior to SEB injection (Hegde et al., 2011). The mice were then treated with RES (100 mg/kg bw) suspended in water by oral gavage in a total volume of 100 μl, 2 h post-SEB injection and then daily until the completion of the experiment. Because SEB is a super antigen, it activates a large proportion of T cells and thus, liver damage is acute and liver enzymes are induced as early as 8 h after SEB, as shown by us previously (Busbee et al., 2015). It is for this reason that we injected RES, 2 h after SEB to test if RES can be used both to treat liver injury induced by SEB. Next, the mice were euthanized either on days 2–3 depending on the nature of experiments as detailed later on. Mice were monitored on daily basis for any signs of distress or any other health-associated problems. In most experiments, we used four groups of mice: Naïve (control), RES only, SEB+Vehicle, and SEB+RES. The experiments were repeated at least three times and each experimental groups consisted of five mice.

### Analysis of Mouse Liver Post-SEB and RES Treatment

Livers were harvested on day 3 post-SEB injection and treatment with VEH or RES. Parts of the livers were stored in 10% formalin for histology and immunohistochemistry and the remaining parts of the livers were used for isolation of MNCs. To examine cell infiltration in liver, fixed liver tissues were embedded in paraffin, 5 μm sections were cut, and then the tissue sections were stained with Hematoxylin and Eosin (H&E). Liver-infiltrating MNCs were isolated from the remaining livers using Percoll density gradient as described earlier (Hegde et al., 2011). In brief, single cell suspensions of livers were prepared using a tissue homogenizer and then passed sterile nylon mesh (70 μM). Cell suspension was washed once with PBS and then the pellet was suspended in 33% Percoll (Sigma-Aldrich) diluted in sterile PBS. The cells in Percoll gradient were centrifuged at 2000 rpm for 15 min at 25°C. MNCs were washed twice with PBS and contaminating RBCs were lysed using RBC-lysis solution (Sigma-Aldrich). Total number of purified MNCs were counted in various treated groups. Purified MNCs were then used for RNA isolation or for staining the cells for various cell markers. For staining MNCs, the cells were first blocked using mouse Fc-block (anti-CD16/CD32) and then stained for various cell surface markers (CD3, CD4, CD8, CD11b, and Gr1) using fluorescently labeled mAb (10 μg/mL, in PBS containing 2% FBS). After washing, stained cells were analyzed in a flow cytometer (FC500, Beckman Coulter). Only live cells were counted by setting gates on forward and side scatters to exclude cell debris and dead cells.

### Analysis of Aspartate Transaminase (AST) Post-SEB and RES Treatment

To assess the liver damage, liver enzyme, aspartate transaminase (AST) was measured by spectrophotometric method at 340 nm in sera collected at 16 h post-treatment. To this end, we used AST assay kit from Pointe Scientific (Canton, MI, United States) as described previously (Hegde et al., 2011; Busbee et al., 2015).

### Characterization of Immune Cells in Spleen Post-SEB and RES Treatment

Spleens from mice exposed to SEB and treated with VEH or RES were harvested on day 3 and single cell suspensions were prepared. The splenic cells were then stained using anti-mouse anti-CD3, anti-CD4, anti-CD8, and anti-NK1.1 monoclonal antibodies (Biolegend, United States). The stained splenic cells were analyzed using flow cytometry (FC500; Beckman Coulter). We also stained splenic cells and liver MNCs with anti-mouse anti-CD11b and -Gr1 to identify myeloid-derived suppressor cells (MDSCs) and analyzed using flow cytometry.

### Analysis of CSF1 in Serum

Enzyme-linked immunosorbent assay (ELISA) was performed to determine the expression of CSF1 levels in the serum samples. To this end, serum was collected from mice 24 h post-SEB+VEH or SEB+RES treatments.

### miR Expression Profiling and Identification of Dysregulated miRs

Total RNAs including microRNAs were isolated from liver MNCs harvested from mice on day 3 post-SEB injection and treatment with VEH or RES. The concentration and purity of the isolated RNAs were determined using a spectrophotometer, and the integrity of the RNA was verified by Agilent 2100 BioAnalyzer (Agilent Tech, Palo Alto, CA, United States). Profiling of miR expression from samples was performed using the Affymetrix GeneChip miRNA 3.0 array platform (Affymetrix, Santa Clara, CA, United States). This array, composed of 2023 miR mouse probes and using the FlashTag biotin HSR hybridization technique, was performed as previously described (Singh N.P. et al., 2012; Guan et al., 2013; Hegde et al., 2013). A heat map was generated by taking the log transformation of fluorescent intensities obtained from the hybridization. Ward’s method was used to carry out hierarchical clustering and similarities were measured using half square Euclidean distance. Fold changes in miR expression were obtained from the array and miRs with > 1.5-fold change were considered for further analysis. Predicted miR targets, alignments, and mirSVR scores were determined by using online miR and miR database (mirwalk v3).

### Real-Time PCR (RT-PCR) to Validate miRs Expression

To validate the expression of miRs (miR-130a and miR-185), quantitative RT-PCR was performed using miScript SYBR green PCR kit and mouse primers for miR-130a- (5′-CAGUGCAAU GUUAAAAGGGCAU; MS00001547) and miR-185-specific (5′-UGGAGAGAAAGGCAGUU CCUGA; MS00001736) from Qiagen (Valencia, CA, United States) were used. SNORD96a (MS00033733) also from Qiagen (Valencia, CA, United States) was used as a positive control for miR expression. The expression level of miR-130a and miR-185 was normalized to SNORD96a levels. Fold change in expression was calculated using the 2^-ΔΔC_T_^ method. Similarly, quantitative real-time PCR (qRT-PCR) was performed to determine the expression of CSF1. We used SsoAdvanced SYBR green supermix from Bio-Rad (Hercules, CA, United States) and mouse CSF1-specific forward (5′-GACCCTCGAGTCAACA GAGC-3′) and reverse (5′-GAGGGGGAAAACTTTGCTTC-3′) primer pairs. Expression level of CSF1was normalized to 18S as described earlier (Singh et al., 2007a,b, 2011).

### Transfection With miR-185 Mimic and Its Inhibitor

We chose miR185 to characterize its role in CSF1 regulation. As shown in Figure 7A, miR-185 has strong binding affinity with complementary sequences of 3′ UTR regions of CSF1 gene. To this end, splenic cells from naïve mice (C57BL/6) were first cultured in complete RPMI 1640 medium at 2 × 10^5^ cells per well in a tissue culture plate and activated with SEB (1 μg/ml). The splenic T cells were transfected using HiPerfect Transfection Reagent kit from Qiagen and following the protocol of the company (Qiagen, Valencia, CA, United States). The cells were transfected with transfection reagents without miR (MOCK) or miR-185 mimic or anti-miR-185 or miR-185 mimic+anti-miR-185 (1:10). Because transfection of primary T cells is difficult, we used activated T cells which are easier to transfect as seen in our previous studies (Alghetaa et al., 2018) leading to transfection rate of > 80–85%. To that end, spleen cells from naïve mice were first activated with SEB for 12 h and then transfection was performed. Such activated cells were transfected with transfection reagents without miR (MOCK) or 20 nM of synthetic mmu-miR-185–5p mimic (5′-UGGAGAGAAAGGCAGUUCCUGA-3′, Cat No: MSY0000214) or anti-mmu-miR-185–5p (5′-UGGAGAGAAAGGCAGUUCCUGA-3′; Cat No: MIN0000214) or combination of miR-185 mimic and anti-miR-185 (1:10). Forty-Eight hours post-transfection, the cells were analyzed for transfection efficiency by flow cytomntry for GFP expression (Positive control). The cells were then treated with VEH or RES (20 μM/ml) for 24 h. The cells were collected and the expression of miR-185 and CSF1 was determined by performing quantitative Real-Time (qRT-PCR) as described above.

### Statistical Analysis

All statistical analysis was performed using GraphPad Prism software (San Diego, CA, United States). Each experimental group had at least five mice. The *in vitro* assays were performed in triplicate. All experiments were repeated at least three times. We used one-way ANOVA to calculate significance and Tukey’s *post hoc* test to analyze differences between the groups, unless otherwise indicated. We used a *p*-value of < 0.05 to determine the statistical significance.

## Results

### Effect of RES on SEB-Induced Acute Liver Injury in Mice

To examine the efficacy of RES on SEB-induced liver injury in mice, SEB was administered into mice (C57BL/6) as described in Section “Materials and Methods.” Mice exposed to SEB were treated with VEH or RES (100 mg/kg body weight). Naïve mice without SEB treatment and mice treated with RES alone were also included as controls.

**FIGURE 1:**
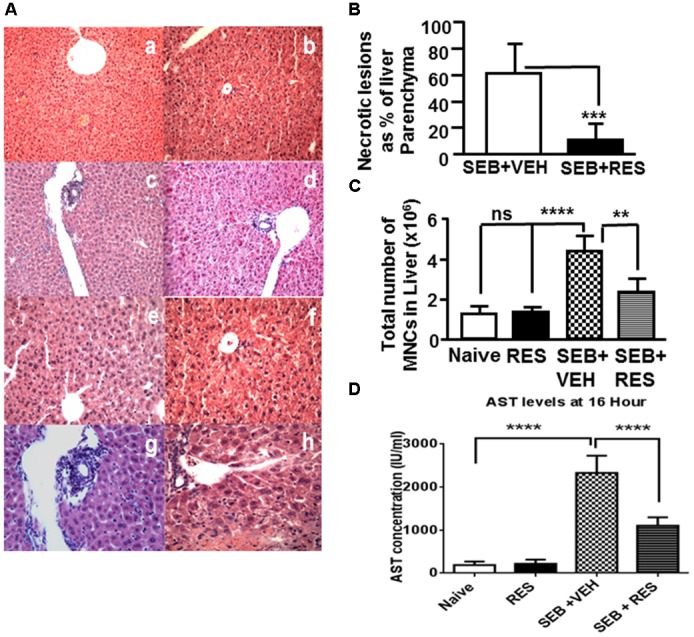
Resveratrol (RES) attenuates SEB-induced liver damage. Mice were untreated (Naïve, Control), treated with RES (100 mg/kg bw) only, SEB+VEH or SEB+RES, as detailed in Section “Materials and Methods.” Livers were harvested, sectioned and H&E staining was performed. **(A)** A representative of H&E stained livers from the four treatment groups; Naïve group (mice with no treatment: **a,b**), RES (mice treated with RES only: **c,d**), SEB+VEH (mice exposed to SEB and treated with VEH: **e,f**), and SEB+RES (mice exposed to SEB and treated with RES: **g,h**). **(B)** Depicts necrotic lesions in the liver of SEB+VEH **(e,f)** SEB+RES **(g,h)**. **(C)** Shows total number of MNCs in liver of various treated groups. **(D)** AST levels in sera. Data presented in **(B–D)** represent mean ± SEM from groups of five mice. Asterisks (^∗^) in **(B–D)** indicate statistically significant (*p* < 0.05) difference between the groups.

Histopathological analysis was performed on liver tissues post H&E staining. The liver tissues from naïve mice showed normal hepatic parenchyma with intact portal triads, hepatic plates of normal thickness, and normal-appearing hepatocytes (Figure 1A,a,b). Similarly, the liver tissue from mice treated with RES alone showed normal hepatic parenchyma with undamaged portal triads and normal-appearing hepatocytes (Figure 1A,c,d), demonstrating no histopathologic evidence of hepatic injury. The liver tissues from mice exposed to SEB+VEH showed marked hepatocyte necrosis (more pronounced in the centrilobular region) affecting approximately 60% of the hepatic parenchyma with relative sparing of the portal triads (Figure 1A,e,f). There were signs of inflammation with focal increase in MNCs (Figure 1A,e,f). In addition, there was an increase in steatosis, when compared to control mice (Figure 1A,e,f). Additionally, the hepatic parenchyma also demonstrated the evidence of hepatic injury with ballooning degeneration and single cell necrosis (Figure 1B). In contrast, in SEB+RES treated mice, the portal triads showed relatively minimal portal triaditis (Figure 1A,g,h). Also, there was less steatosis in the liver tissues of SEB+RES mice (Figure 1A,g,h). Although inflammation was observed in the liver SEB+RES mice, the level of inflammation was significantly reduced, when compared to liver tissues from mice treated with SEB+VEH (Figure 1A,g,h). The liver tissues from mice exposed to SEB+RES showed significant decrease in centrilobular necrosis affecting approximately 10% of the hepatic parenchyma when compared to ∼60% in SEB+VEH groups (Figure 1B). Next, we studied the total number of purified liver MNCs. There was no significant change between Naïve and RES groups (Figure 1C) but there were significantly higher number of MNCs in livers from SEB+VEH-treated mice (Figure 1C), when compared to VEH- or RES-treated groups. In contrast, there was significantly less number of MNCs in SEB+RES-treated groups, when compared to SEB+VEH group (Figure 1C).

Upon examination of liver enzyme aspartate transaminase (AST) levels in sera, we noted minimal AST levels (<100 IU/L) in both naïve and RES alone treated mice (Figure 1D). However, SEB+VEH-treated mice showed significant increase (more than 2000 IU/L) in AST levels (Figure 1D), while mice exposed to SEB+RES showed significant decrease (∼1000 IU/L) in AST levels (Figure 1D). These data together demonstrated significant attenuating effect of RES on SEB-induced acute liver injury.

### RES Affects Immune Cell Populations of Both Spleen and Liver

**FIGURE 2:**
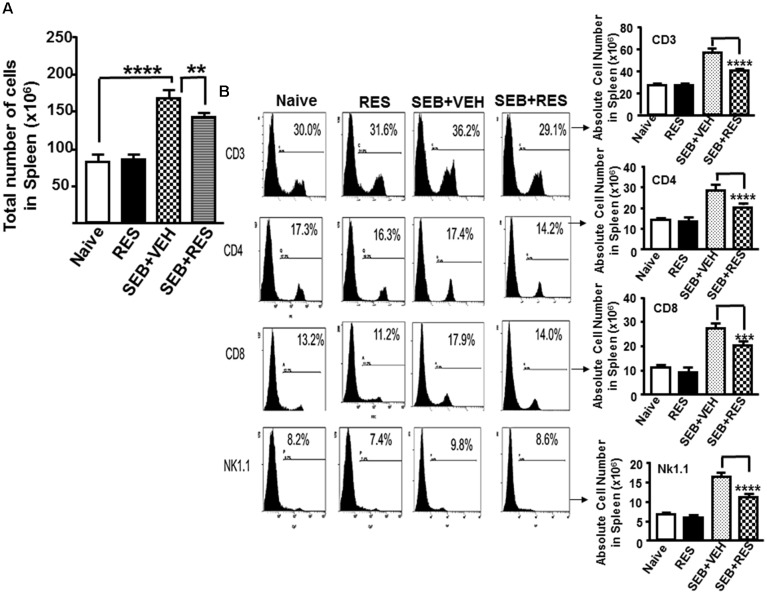
Characterization of immune cells in mice with acute liver injury. Acute liver injury was induced as described in Figure 1 legend. Spleen cells isolated from livers were further analyzed. **(A)** Shows total number of cells in spleen. **(B)** Spleen cells were stained with fluorophore-labeled anti-mouse anti- CD3, -CD4, -CD8, and -NK1.1 antibodies. The data are presented as percentage of various subsets of cells as well as total number of individual population of such cells in the spleen. Vertical bars represent mean ± SEM from groups of five mice. Asterisk (^∗^) indicates statistically significant (*p* < 0.05) difference between groups.

Next, we examined the effect of RES on immune cells in the spleen as well as in the liver. There was a significant increase in total number of immune cells in spleen (Figure 2A) in mice exposed to SEB+VEH, when compared to mice that received either none (Naïve; control) or treated with RES alone. In contrast, there was a significant reduction in the number of immune cells in spleen (Figure 2A) of mice that were exposed to SEB+ RES, when compared to SEB+VEH group (Figure 2A).

Upon examination of the presence of various immune cells in the spleen and liver using various cell markers (CD3, CD4, CD8, and NK1.1), we noted no significant change in the total number of CD3+, CD4+, CD8+, and NK1.1+ cells in control and RES alone-treated groups of mice (Figure 2B). However, there was significant increase in total number of all the four cell populations in mice that were exposed to SEB+VEH, when compared to control or RES-treated mice (Figure 2B). Moreover, there was a significant decrease in cell populations of all the four cell types examined in mice exposed to SEB+RES, when compared to mice that received SEB+VEH (Figure 2B). These data demonstrated that SEB, being a super antigen induces proliferation of large numbers of T and NK1.1 cells while RES can suppress the induction of such cells.

### RES Promotes Generation of Immunosuppressive MDSCs

**FIGURE 3:**
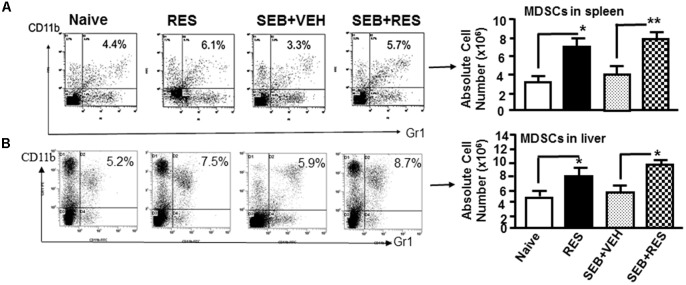
Resveratrol induces MDSCs in the spleens and liver. Acute liver injury was induced as described in Figure 1 legend. MDSCs were stained for the expression of Gr1 and CD11b. The percentage and total number of MDSCs in the spleen **(A)** and in the liver **(B)** is shown. Vertical bars in represent mean ± SEM using groups of five mice. Asterisk (^∗^) indicates statistically significant (*p* < 0.05) differences between the groups.

Next, we analyzed for CD11b+/Gr1+ MDSCs cells by flow cytometry because previous studies from our laboratory have shown that RES induces MDSCs that are highly immunosuppressive (Singh U.P. et al., 2012). The data showed that there was no significant change in the percentage and absolute numbers of MDSCs in the spleens of naïve, RES only and SEB+VEH groups of mice tested. Interestingly, in SEB+RES group, there was a significant increase in the total numbers of MDSCs when compared to SEB+VEH treated group (Figure 3A). Upon analysis of MDSCs in liver MNCs, we noted significant increase in MDSCs in RES-treated group, when compared to Naïve control (Figure 3B). However, there was no significant change in MDSCs in SEB+VEH group, when compared to naïve control. Moreover, there was a significant increase in total number of MDSCs in liver of SEB+RES group when compared to SEB+VEH and Naïve groups (Figure 3B). These data suggested that RES might suppress SEB-mediated inflammation through induction of MDSCs.

### RES Alters the miR Profile in Liver-Infiltrating MNCs

**FIGURE 4:**
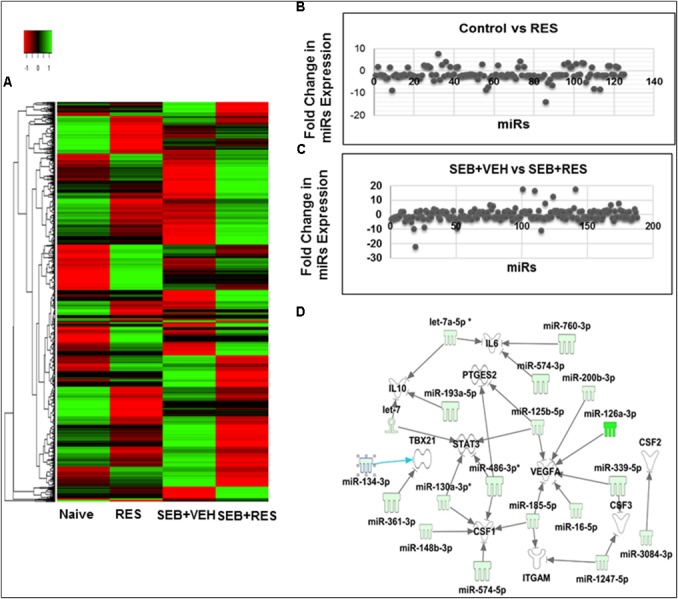
Heat map of miR expression in liver MNCs in mice with acute liver injury and Pathway Analysis of dysregulated miRs in liver MNCs. Acute liver injury was induced as described in Figure 1 legend. miR profile in liver MNCs was performed by using miR arrays on Affymetrix CGS300 System (Affymetrix). **(A)** Heat map depicting miR expression profile in liver MNCs of mice in the four groups. The expression pattern (green to red) represents the spectrum of downregulated to upregulated expression pattern of miRs, respectively. **(B)** Shows differential expression of miRs between Control and RES groups. **(C)** Shows differential expression of miRs between SEB+VEH and SEB+RES groups. **(D)** Dysregulated miRs in liver MNCs post-RES treatment in acute liver injury-induced mice were analyzed using Ingenuity pathway analysis (IPA) software online (Qiagen). There was a direct relationship of dysregulated miRs and several expected target genes including CSF gene families (CSF1, CSF2, CSF3), as well as VEGFA, PTGES2, STAT3, TBX21, and IL-10.

We next examined the miR profile in the liver-infiltrating MNCs cells of mice exposed to SEB and treated with VEH or RES. We performed cluster analysis of 2023 miRs (Figure 4A) using Ward’s method and as defined by median absolute deviation in SEB+VEH and SEB+RES. We further measured the expression of miRs in the two groups using Half Square Euclidean Distance method. Ordering function of miRs was done based on input rank. Comparison of control vs. RES revealed 95 downregulated and 30 Upregulated miRs, while comparison of SEB+VEH vs. SEB+RES groups revealed 105 downregulated miRs and 74 upregulated miRs with a fold change of > 1.5 fold (Figures 4B,C).

### RES-Regulated miRs Play Important Role in the Expression of CSF Family Genes

To understand the role of RES-mediated alterations in miRs in the regulation of genes that participate in anti-inflammatory responses, we analyzed some of the downregulated miRs using IPA software and the database of the company (Qiagen). These miRs were shown to target various genes including CSF1, CSF2, CSF3, VEGFA, PTGES2, STAT3, TBAX21 and IL-10 genes (Figure 4D). The CSF1 gene was particularly interesting because it has been shown to play a key role in the induction of MDSCs (Kumar et al., 2017), and we observed significant induction of MDSCs following RES treatment (Figures 3A,B).

### Validation of miR Expression by Real-Time PCR

**FIGURE 5:**
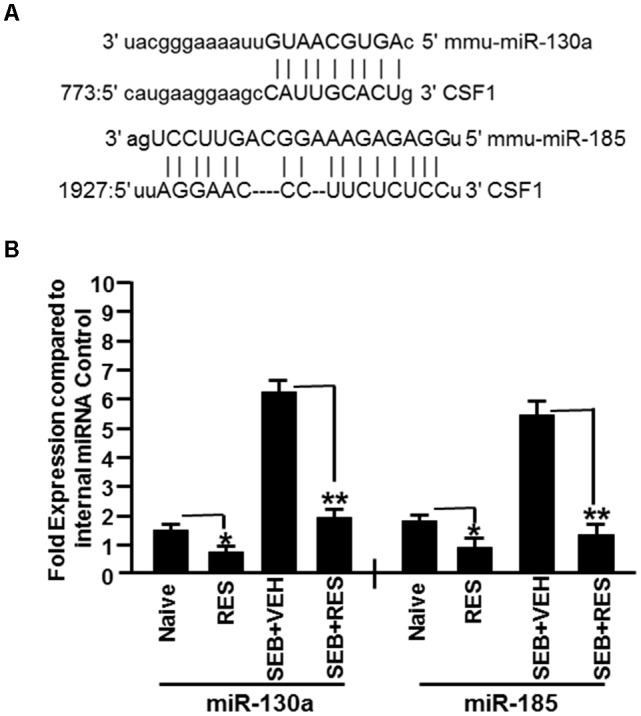
Validation of expression profile of selected miRs in liver MNCs. We selected two miRs (miR-130a and miR-185) based on their ability to target CSF1 as suggested in figure for further validation. **(A)** Seed sequence and alignment of the miR-130a and miR-185 with 3′ UTR of CSF1 gene. **(B)** Using mouse miR-specific primers for mature miR-130a and miR-185, Real-Time PCR was performed to determine their expression in liver MNCs. SNORD96a was used as an internal control. Data presented for both miR-130a and miR-185 represent the mean ± SEM of three independent experiments **(B)**. Asterisk (^∗^) indicates statistically significant (*p* < 0.05) difference between the groups compared.

Based on the analysis of miRs array data, we selected two downregulated miRs that targeted CSF1 (miR-130a and miR-185) (Figure 5A) to verify and validate their expression in liver MNCs harvested from naïve, RES, SEB+VEH, and SEB+RES groups of mice. We performed quantitative Real-Time PCR using cDNA generated from total RNAs including miRs isolated from MNCs of the four groups. Real-Time PCR data demonstrated significant downregulation in the expression of miR-130a and miR-185 in RES only treated mice, when compared to control mice (Figure 5B). Interestingly, SEB+VEH group showed robust upregulation of miR-130a and miR-185 when compared to the control group while SEB+RES group showed significant downregulation in the expression of these miRs (Figure 5B). Thus, the data obtained from Real-Time PCR validated the expression profile of miR-130a and miR-185 obtained from miR-array and demonstrated that SEB induces these miRs while RES decreases their expression.

### RES Upregulated the Expression of CSF1 in Liver MNCs

**FIGURE 6:**
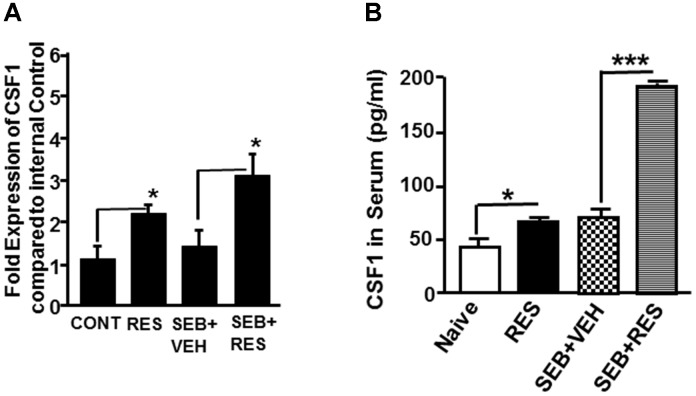
Expression of CSF1 in liver MNCs and serum. Expression of CSF1 was measured in the four groups of mice that are described in Figure 1 legend. **(A)** Expression of CSF1 gene in liver MNCs. Expression of mouse CSF1 gene in liver MNCs was determined by performing Real-Time quantitative PCR. Mouse CSF1-specific primers were used to perform Real-Time PCR on cDNAs generated from total RNAs isolated from liver MNCs of the four groups (naïve/control, RES, SEB+VEH, and SEB+RES). The internal control consisted of 18S. Data presented for CSF1 gene represent the mean ± SEM of three independent experiments and asterisk (^∗^) indicates statistically significant (*p* < 0.05) difference between the groups. **(B)** Measurement of CSF1 levels in the sera of mice using ELISA. Vertical bars represent data with mean ± SEM of groups of five mice and asterisks (^∗^) indicate statistically significant (*p* < 0.05) difference between groups compared.

Because CSF1 has been shown to play a key role in the induction of MDSCs (Kumar et al., 2017), and miR studies revealed down-regulation of miR-130a and miR-185 that target CSF1, we determined the expression of CSF1 in liver MNCs by quantitative Real-Time PCR. The data showed that there was a significant increase in the expression of CSF1 gene in RES only treated mice as well as SEB+RES groups when compared to control mice or those treated with SEB+VEH (Figure 6A). Additionally, we measured the levels of G-CSF in the sera of these mice and found that treatment with RES alone or SEB+RES significantly increased the G-CSF levels in the serum (Figure 6B). These data together demonstrated that RES significantly increases the expression of CSF1 in liver MNCs and G-CSF in the serum.

### Analysis of miR-185-Associated CSF1 Expression

**FIGURE 7:**
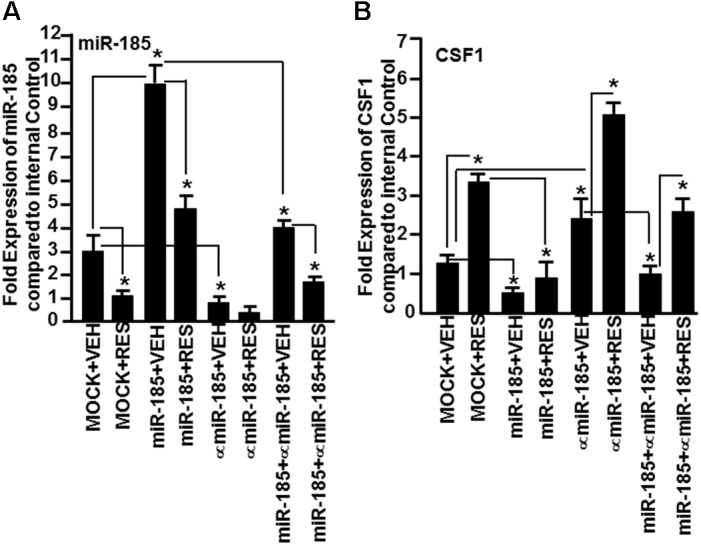
Demonstration that miR-185 targets CSF1. Splenic T cells were transfected with MOCK (all transfection reagents without miR-185 mimic) or with mature miR-185 or anti-miR-185 or miR-185+anti-miR-185 (1:10 concentration). Forty-eight hours post-transfection, the cells were activated with SEB and treated with VEH or RES for 24 h. Expression of **(A)** miR-185 and CSF1 **(B)** genes was determined by performing Real-Time PCR. Data of Real-Time PCR are presented as fold change in expression relative to internal control (SNORD96a) for miR-185 or 18S for CSF1 gene. Data are depicted as mean ± SEM of three independent experiments and asterisks (^∗^) indicate statistically significant (*p* < 0.05) difference between groups compared.

Because miR-185 showed strong binding affinity to CSF1 gene (mirwalk v3 and TargetScan), we investigated whether miR-185 plays a direct role in regulating the expression of CSF1 gene in mice. To that end, we transfected primary T cells with mature miR-185 mimic or anti-miR-185 or both, and cultured in the presence of SEB. Forty-eight hour post-transfection, the transfected T cells, were cultured in the presence of VEH or RES for 24 h. Next, we determined the expression of miR-185 and CSF1 by performing Real-Time PCR. T cells not transfected with miR-185 showed moderate miR-185 expression but RES significantly downregulated the expression of miR-185 (Figure 7A). In contrast, T cells transfected with miR-185 showed significantly higher expression of miR-185 but its expression was significantly downregulated in the presence of RES (Figure 7A). T cells transfected with anti- miR-185, on the other hand, showed significantly downregulated miR-185 expression and its expression was further decreased in the presence of RES (Figure 7A). As suggested by the protocol of the manufacturer (Qiagen), T cells transfected with both miR-185 and anti-miR-185, showed significantly downregulated expression of miR-185 and RES further downregulated the expression of miR-185 (Figure 7A). These transfection studies confirmed that RES downregulates the expression of miR-185 in T cells.

We, next, examined the expression of CSF1 in T cells not transfected or transfected with miR-185 or anti-miR-185 or both, and treated with VEH or RES. As shown in Figure 7B, there was a moderate expression of CSF1 in VEH-treated untransfected T cells but CSF1 expression was significantly higher in T cells post-RES treatment (Figure 7B). However, CSF1 expression in T cells transfected with miR-185 significantly decreased, when compared to VEH-treated untransfected T cells (Figure 7B). Upon treatment with RES, there was further downregulation of CSF1 in T cells, when compared to T cells MOCK transfected and treated with VEH or RES (Figure 7B). T cells transfected with anti-miR-185 and treated with VEH, on the other hand, showed a significant increase in CSF1 expression, when compared to VEH-treated untransfected T cells (Figure 7B). Furthermore, RES treatment of anti-miR-185 transfected T cells further significantly increased CSF1 expression (Figure 7B). When T cells were transfected with both miR-185 and anti-miR-185, CSF1 expression was downregulated, when compared to T cells transfected with anti-miR-185 (Figure 7B). Moreover, RES treatment significantly reversed the expression of CSF1 (Figure 7B). Taken together, these data suggested that RES decreases the expression of miR-185 which targets CSF1 thereby leading to induction of CSF1 expression.

## Discussion

*Staphylococcus aureus* is a ubiquitous Gram-positive bacteria that is found to colonize about one-third of the general population. It is an opportunistic pathogen that triggers infections through contaminated food. The enterotoxins such as SEB produced by these bacteria act as super antigens by way of activating a large proportion of T cells, triggering cytokine storm, acute toxic shock, multi-organ failure and mortality (McKallip et al., 2005; Krakauer et al., 2016). In the current study, we investigated whether RES, which is well-known for its potent anti-inflammatory property, can protect SEB-induced inflammation in liver and attenuate acute liver injury.

In the current study, we demonstrated that the administration of SEB caused an increase in AST levels as well as necrotic lesions in the liver. Additionally, the number of immune cells consisting of CD3+, CD4+ or CD8+ T cells, and NK1.1 cells, increased significantly in both the livers and spleens following SEB administration. RES treatment in SEB immunized mice caused an increase the population of MDSCs in the spleen as well as in the liver. Moreover, RES treatment led to reversal of all such inflammatory indicators and the livers were significantly protected from SEB-mediated injury. These data are consistent with our previous studies that RES is a potent anti-inflammatory agent that can protect mice from acute lung injury mediated by SEB (Rieder et al., 2012; Alghetaa et al., 2018), and extend these data by identifying the miR that regulates CSF1, which is involved in MDSC induction.

Myeloid derived suppressor cells are well-characterized by our lab and elsewhere as potent immunosuppressive cells (Gabrilovich et al., 2001; Hegde et al., 2008, 2013; Gabrilovich and Nagaraj, 2009; Singh U.P. et al., 2012). MDSCs have been well-studied and characterized in recent years in various tumor models as well as in cancer patients (Fujimura et al., 2010) for their immunosuppressive functions. In general, MDSC numbers increase significantly during cancer development, which in turn leads to suppression of anti-tumor immunity and enhanced tumor growth (Schmid and Varner, 2010). Hammerich and Tacke (2015) reported that the liver is a primary site of MDSCs *in vivo* and suggested that modulating the functionality of MDSCs might represent a promising therapeutic target for liver diseases (Hammerich and Tacke, 2015). MDSCs use several mechanisms to trigger immunosuppression, such as production of arginase I and inducible nitric oxide synthase (iNOS), leading to inhibition of T cell proliferation. However, more recently, it has been shown that MDSCs may also be induced at sites of inflammation and may prevent tissue injury by downregulating T cell responses (Bronte and Mocellin, 2009). An earlier study demonstrated that repeated systemic administration of staphylococcal enterotoxin A (SEA) led to induction of tolerance via accumulation of MDSCs in the spleen, and this process was IFN-γ dependent (Cauley et al., 2000). We have also shown that RES attenuated chronic colitis in IL-10 knockout mice through MDSC induction (Singh U.P. et al., 2012). Also, RES was shown by our laboratory to induce immunosuppressive MDSCs in a lung-injury model (Rieder et al., 2012). In general, MDSCs get activated and expand in the presence of pro-inflammatory cytokines and other mediators such as G-CSF, CSF1 (M-CSF), GM-CSF, IL-1β, IL-12, and IFN-γ (Gabrilovich and Nagaraj, 2009). In the current study, we used miR pathway analysis to identify potential miRs that may trigger CSF1, a cytokine known to induce MDSCs. Interestingly, such studies led to identification of miR-130a and miR-185 that were found to target CSF1. These data also suggested that CSF1 induction by RES may play a critical role in MDSC induction inasmuch as previous studies showed that blocking CSF1 prevents the generation of MDSCs (Holmgaard et al., 2016).

Since the discovery of miRs, about a decade or so, these non-coding endogenous small RNAs have been shown to play a major role in the regulation of the immune responses (Pedersen and David, 2008), including autoimmunity and inflammation (Singh et al., 2013). Recent studies from our lab and elsewhere have shown that miRs play a critical role in promoting an anti-inflammatory state (Singh et al., 2013, 2014, 2016; Zhou et al., 2014; Guan et al., 2016). In the current study, therefore, we investigated if treatment with RES following SEB injection would alter the expression of miRs in the immune cells. The data demonstrated that RES treatment led to altered expression of a significant number of miRs in liver infiltrating MNCs of mice. Pathway analysis of miRs that are down-regulated by RES led to identification of two miRs (miR-130a and miR-185) that had strong binding affinity with complementary sequences of 3′ UTR regions of CSF1 gene. Because RES downregulated the expression of both miR-130a and miR-185, these data suggested the mechanisms through which RES may increase CSF1 expression. The role of miR-185 to regulate CSF1 expression was further confirmed by performing transfection experiments. There was significant downregulation of CSF1 expression in T cells that were transfected with miR-185 mimic while the expression of CSF1 was significantly upregulated in T cells that were transfected with anti-miR-185. These data also correlated with significant induction of CSF1 in liver MNCs and serum G-CSF concentrations in SEB+RES group when compared to SEB+VEH.

This is the first study that demonstrates the role of miR-185 in the regulation of CSF1. miR-130a and miR-185 have been shown to regulate other cytokines. For example, IL-10Rα has been shown to be directly regulated by miR-185 (Venza et al., 2015). Interestingly, miR-130a-3p was shown to target transforming growth factor-beta receptors (TGFBRs) 1 and 2. Thus, overexpression of miR-130a-3p in hepatic stellate cells (HSCs) inhibited their activation and proliferation, with the decreased expression of TGFBR1 and TGFBR2. TGF-β has been shown to control the generation of MDSCs (Lee et al., 2018). Thus, RES-mediated decrease in miR-130a may enhance TGF-β pathway to induce MDSCs. Woo et al. (2013) have shown that miR-130a regulates CSF1 mRNA decay in ovarian cancer cell demonstrating a role for miR-130a in CSF1 regulation.

In summary, the current study suggests that RES attenuates SEB-induced liver injury through modulation of miRs. Because SEB is a super antigen that drives cytokine storm, our studies suggest that RES is a potent anti-inflammatory agent that has the potential to be used as a therapeutic modality to treat acute inflammation triggered by bacterial enterotoxins. Our studies also suggest that RES may mediate its effects through alterations in the expression of miR-130a and miR-185, which target CSF1 expression and consequently trigger highly immunosuppressive MDSCs.

In addition to the immunosuppressive properties exerted by RES against SEB, a bacterial enterotoxin, as shown in the current study, RES has also been shown to exert antibacterial activity (Ma et al., 2018). Also, RES may act an antagonist, when used in combination of antimicrobial agents. Recently, Tosato et al. (2018) have shown that the antimicrobial capacity of levofloxacin or photodynamic therapy was significantly diminished when levofloxacin or methylene blue were co-administered together with RES, indicating that consumption of RES during antimicrobial treatment should be cautioned (Tosato et al., 2018). In another study, Liu et al. (2016) showed that RES antagonizes antimicrobial lethality and stimulates recovery of bacterial mutants. In the light of these reports, one should be cautious to use RES when antibiotic agents are being used to treat pathogenic bacteria. However, we also wish to add that our studies have focused on SEB which can also be used directly as a bioterrorism agent to cause toxicity and organ failure, and that in such instances, RES as well as miRNA identified may serve as a treatment modality.

## Author Contributions

SK, NS, PN, and MN conceptualized and designed this work. SK and NS were involved in sample processing and data collection, conducted the primary investigation and statistical analysis of the data, and wrote the manuscript. PN and MN provided resources, analysis tools, and responsible for funding acquisition. SK, NS, EZ, TC, SC, LH, PN, and MN reviewed and edited the manuscript. AA contributed to the design of one experiment and reviewed the manuscript. All authors approved the final manuscript.

## Conflict of Interest Statement

The authors declare that the research was conducted in the absence of any commercial or financial relationships that could be construed as a potential conflict of interest.
